# A local-authority specific definition of research: Results from a Delphi study

**DOI:** 10.1016/j.puhip.2026.100765

**Published:** 2026-03-04

**Authors:** L.M. Brown, S. Sowden, A.D.M. Briggs, A. Dennington-Price, V. Dutta, S. Hampshaw, E. Humphreys, T. Johns, A. Levitas, R. Murphy, K. Needham, L. Renwick, L. Hayes

**Affiliations:** a NIHR Research Support Service Specialist Centre for Public Health, Newcastle University, Newcastle Upon Tyne, UK; b Oxfordshire County Council, Oxford, UK; c NIHR Research Support Service Specialist Centre for Public Health, University of Southampton, Southampton, UK; d Patient & Public Contributor, UK; e Slough Borough Council, Slough, UK; f NIHR Health Determinants Research Collaboration (HDRC) Doncaster, City of Doncaster Council, Doncaster, UK; g NIHR Health Determinants Research Collaboration (HDRC) Tower Hamlets, London Borough of Tower Hamlets, London, UK; h Institute of Public Health and Wellbeing, University of Essex, Colchester, UK; i London Borough of Islington, London, UK; j North Yorkshire Council, Northallerton, UK; k NIHR Health Determinants Research Collaboration (HDRC) North Yorkshire, Northallerton, UK

**Keywords:** Local authority, Research, Delphi, Evidence

## Abstract

**Objectives:**

There is an increasing focus on promoting evidence informed decision making within local government. However, there is a lack of consistency within this setting on how research is defined in the context of evidence for decision making. This lack of consistency presents a challenge for local authority officers in identifying and communicating about research activities and in establishing appropriate research governance and ethics processes.

This study aimed to develop a consensus definition of research in a local authority setting.

**Study design:**

A Delphi method was used.

**Methods:**

Potential definitions of local authority research were identified by the project Steering Committee. These were circulated to the Delphi panel, comprising 60 local authority officers, via an online Round One questionnaire. Round One definitions that achieved high levels of agreement (>75%) from the panel were revised and included in a Round Two questionnaire, circulated to the same panel. Responses from Round Two were taken to an in-person workshop of the project Steering Committee where a consensus definition, reflecting the statements with the highest levels of agreement, was developed.

**Results:**

After two rounds of online questionnaire (with response rates of 80% and 77% respectively), four definition statements which had the highest level of agreement were identified by the Steering Committee for inclusion within the final consensus definition. The format and exact content of the definition was finalised by the Steering Committee to reflect key components of the purpose of research and its process, which were identified as being important by the Delphi panel.

**Conclusions:**

Our definition of local authority research has potential to support the development of a research culture in local authorities. It provides a basis for communicating about research, identifying opportunities for research and for establishing appropriate ethics and governance processes.

## 1 Introduction

Decisions made in local authorities impact population health and health inequalities by influencing policy, priority setting and commissioning relevant to the wider determinants of health. This includes, for example, decisions around housing, transport and education related activity [1].

Local authorities operate within unprecedented financial constraints. The recent increase in the Public Health Grant of 3% in real terms does little to address the 20% decrease imposed between 2016 and 2023 [2]. Therefore, it is essential that local authority decision makers use research evidence to inform their policy and commissioning decisions to use their resources to maximum effect [3].

Despite the acknowledgment that local authority decisions should be evidence informed, demonstrated by the considerable National Institute for Health and Care Research (NIHR) investment in Health Determinants Research Collaborations (HDRCs) to facilitate this [4], local authorities, and indeed departments within local authorities, are at very different stages in terms of their research readiness [5].

In the NHS, an established research infrastructure exists underpinned by a clear definition of research. In local authorities the research infrastructure is nascent. Recent work highlighted how a lack of consistency in how research is defined in local authorities is a barrier to developing a research culture [6]. Existing definitions of research used in other settings (e.g. NHS or universities) are narrow in focus and don't reflect the multidimensional aspects of research activities in local authorities [7]. Increasing evidence-informed practice in heterogenous local authorities that are not uniform in the extent to which they engage with research evidence is challenging. It requires a collective understanding of how research in this context is defined and what the requirements of decision makers are in relation to research evidence and how it is communicated [3].

Our aim was to achieve consensus on a definition of research in a local authority setting.

## 2 Methods

We used a Delphi method [8]. This structured, iterative technique for generating consensus amongst a group of individual experts (Delphi panel) is commonly used within health sciences [9].

Key features of the Delphi method that made it appropriate for this work are that individual panel members remain unknown to each other, minimising bias that can arise from group meetings and it allows opinions from a range of individuals who are geographically diverse to be sought [9].

**Fig. 1:**
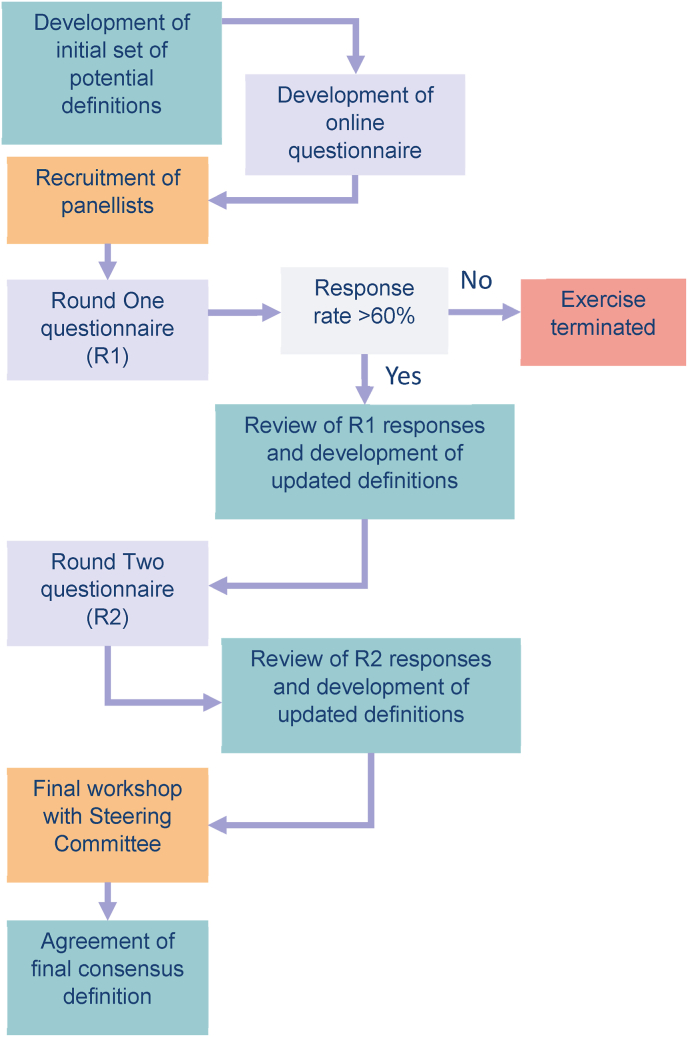
Flowchart of the Delphi process.

We conducted two rounds of online questionnaires, followed by an in-person workshop with the Steering Committee. Fig. 1 illustrates this process. The number of questionnaire rounds was limited as panellist attrition and false consensus are risks of having more rounds [10].

### 2.1 Steering Committee

We convened a Steering Committee comprising local authority officers, practitioner-academics, academic researchers and a public representative. Steering Committee members were involved in agreeing the definitions of research for the first questionnaire round, piloting online questionnaires, reviewing and revising definitions between rounds, and in the final workshop.

### 2.2 Recruitment and selection of Delphi panellists

Panellists were recruited from local authorities between July and September 2024. The eligibility criterion was current employment within a UK local authority. No restrictions were placed on job role, department or directorate type, or length of experience.

**Fig. 2:**
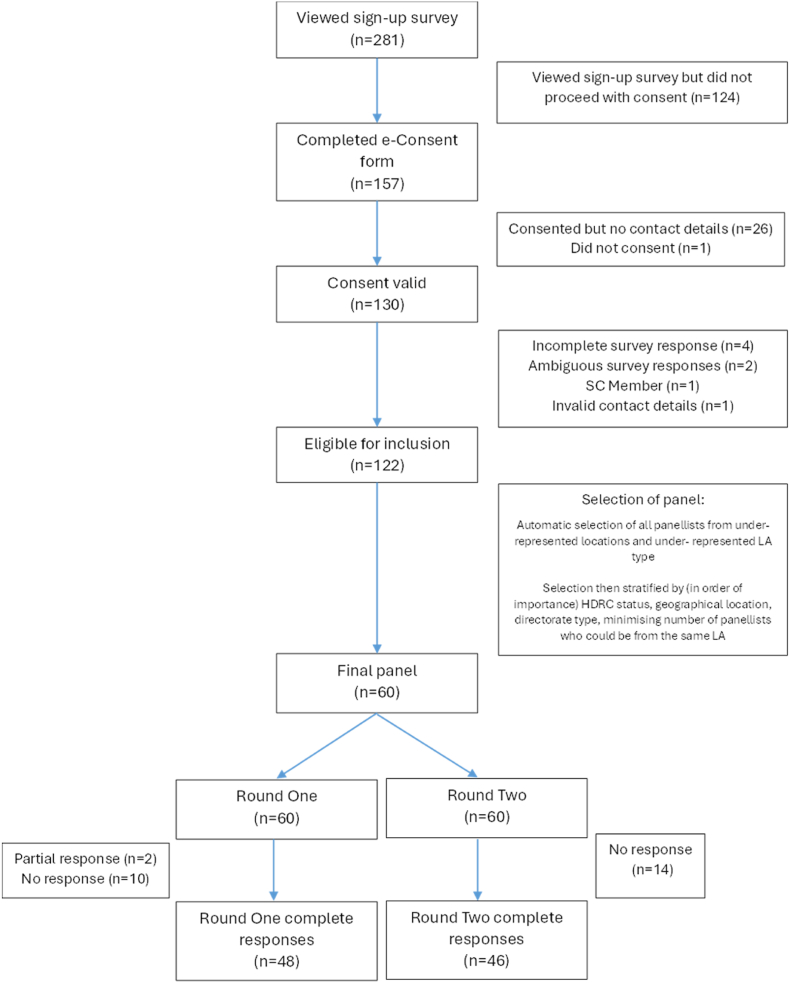
Summary of recruitment and responses.

One hundred and thirty potential panellists initially expressed an interest from whom a panel of 60 were selected. Panellist number was limited to 60 to minimise difficulties associated with larger panels including lower response rates and data management issues [11]. Panel selection ensured representation across UK regions, different local authority types (e.g. Tier 1 or Tier 2), between local authorities with and without Health Determinants Research Collaboration (HDRC) status, and across directorate types. Fig. 2 summarises the recruitment process. Characteristics of the panel are provided in Table 1.

**Table 1:** Characteristics of the Delphi panel.

Local authority region		n	%

	East of England	5	8
	East Midlands	4	7
	London	7	12
	North East	6	10
	North West	7	12
	Scotland	2	3
	South East	9	15
	South West	5	8
	West Midlands	7	12
	Yorkshire and the Humber	8	13

Local authority type		n	%

	Tier 1 (Metropolitan, County, Unitary or London Borough)	54	90
	Tier 2 (District)	6	10

Directorate type		n	%

	Communities	3	5
	Corporate/Central	11	18
	Other	10	17
	Public Health	31	51
	Social Care	4	7
	Planning/Regulatory	1	2

HDRC status		n	%

	HDRC	29	48
	Non-HDRC	19	32
	Unsure	12	20

Type of role		n	%

	Director/Head of a Service or equivalent	10	17
	Entry-Level Management	11	18
	Mid-Level Management	16	27
	Other	11	18
	Senior/Top Level Management	12	20

Local authority experience		n	%

	Less than 1 year	5	8
	1-5 years	21	35
	5-10 years	13	22
	10-15 years	10	17
	15-20 years	5	8
	More than 20 years	6	10

Research experience		n	%

	Current role with no links to research, and no previous experience/links	1	1
	Current role with no links to research but previous experience/links	3	5
	Current role with awareness of ‘research’ but no direct involvement	5	8
	Current role linked to research	46	77
	No LA research experience, but previous research experience in other organisation	4	7
	Previous LA research experience, but now in a different non-research role	1	2

### 2.3 Development of initial definitions

The list of initial definitions which formed Round One of the Delphi comprised published definitions of research [12,[13], [14], [15], [16]]. Definitions suggested by the Steering Committee based on their expertise were also incorporated. The list of definitions was revised iteratively by the Steering Committee until agreement was reached. This resulted in 24 initial definitions (see Table 2).

**Table 2:** Round One definitions of research with Round One response (agree or disagree) percentages.

Potential definition	% agree	% disagree
Research includes any activity that assesses a novel intervention by randomly allocating participants to receive the intervention or an alternative.	82%	18%
Research includes any activity that involves a systematic investigation designed to develop or contribute to generalisable knowledge.	90%	10%
Research includes any activity that addresses a question with scientifically sound (and reproducible) methods and has clearly defined aims and objectives.	96%	4%
Research includes any activity that involves collecting additional data to that collected routinely.	52%	48%
Research includes any activity that uses existing, routinely collected data (secondary data) in a new way to provide insights and guide new activity.	79%	21%
Research includes any activity that involves the systematic collection, analysis, and interpretation of data relating to an area of focus (e.g. the wider determinants of health).	84%	16%
Research includes any activity that involves evaluating existing, already in use, interventions or services with the aim of reviewing, improving or developing them (including judging how well a service is performing, possibly against a predetermined standard).	73%	27%
Research includes any activity that includes evidence syntheses activities (bringing together data from multiple sources to provide a summary of existing knowledge) e.g. systematic review, meta-analysis.	96%	4%
Research includes any activity that may be co-designed and co-produced with the local community/service users.	63%	38%
Research includes any activity that involves administering a questionnaire to, or using other methods (e.g. focus groups) to gather the views of, the public, service users or staff on a particular subject, service, or issue.	75%	25%
Research includes any activity that involves collecting information on the views and experiences of a defined population e.g. residents of a specific town, city or region.	75%	25%
Research includes any activity that has a primary aim of producing or contributing to generalisable or transferable new knowledge to answer or refine relevant questions using scientifically sound methods.	96%	4%
Research includes any activity that aims to ask a question which has not yet been answered (i.e. to tell us something new) and which may be used as the basis for decision making.	83%	17%
Research includes any activity that focuses on understanding population behaviour and issues, considering factors (e.g. socioeconomic, cultural, environmental) of influence to improve health and wellbeing.	88%	13%
Research includes any activity that aims to produce findings to inform policy and what interventions should be invested in, for example to improve population health and wellbeing and reduce health inequalities.	92%	8%
Research includes any activity that has the primary intention of preventing disease or injury or improving an existing programme or service.	46%	54%
Research includes any activity that will potentially save LAs money, or allow LA budgets to be more accurately targeted, via new approaches or insights which will allow efficiency savings to be made.	67%	33%
Research includes any activity that generates information for internal use only.	23%	77%
Research is any activity that aims to generate a publishable output (e.g. academic journal article).	67%	33%
Research includes any activity that aims to produce an output that could be of use or interest beyond the immediate service/area where the activity is taking place.	69%	31%
Research includes any activity that intends to identify ways to improve the wellbeing, health or efficiency of people employed by, or working with, the LA.	65%	35%
Research includes any activity that involves collecting data with, or on behalf of, an academic institution (e.g. a university) including for the purpose of obtaining a formal qualification.	67%	33%
Research includes any activity that is externally funded via a research funding body, charity research funding stream or commercial entity.	73%	27%
Research includes any activity that falls outside of, or goes beyond, routine practice/business as usual and that may represent a risk to individuals, or the organisation, if not managed appropriately.	42%	58%

### 2.4 Round One questionnaire

The 24 initial definitions were circulated to panellists via an online questionnaire (which remained open for three weeks to allow sufficient time for panellist response) in September 2024. Panellists reviewed each definition and indicated if they agreed or disagreed with it. The survey was designed so that each panellist was presented with the definitions in a random order to minimise the chance of response fatigue introducing bias. Panellists could comment against each definition and suggest their own definition.

As high response rates are essential in a Delphi study [17], a minimum response rate of 60% was set for Round One.

### 2.5 Round Two Questionnaire

Definitions from Round One that at least 75% of respondents agreed with were included in the Round Two Questionnaire, circulated for three weeks in November 2024. The threshold for inclusion for Round Two was based on commonly reported thresholds within this methodology [18] and previous Delphi projects in a similar setting [19]. Panellists’ comments and suggested definitions from Round One were thematically analysed to inform the definitions taken through to the next round which were reviewed and agreed by the Steering Committee prior to circulation in Round Two.

In Round Two panellists were reminded about the purpose of the project and given instructions on what to consider. The guidance emphasised that this Delphi intended to achieve consensus on what research in a local authority setting is and that considerations of whether something needed ethical, or research governance, review should not influence panellists’ decisions.

Round Two definitions were presented with an explanation of how they were derived from Round One and the level of agreement achieved for each. Examples of hypothetical local authority research projects to help illustrate each Round Two definition were also included. These examples were devised by the Steering Committee.

In Round Two, panellists were asked to indicate their level of agreement with each definition using a Likert scale. They were also able to add comments. It is common to use a Likert scale in Delphi studies [18] and was appropriate at this stage as there were fewer definition statements than Round One.

Two definitions of what research is not (devised by the Steering Committee based on professional experience) were included within Round Two in response to comments made by panellists and discussion by the Steering Committee indicating that this would be helpful.

At the end of the questionnaire, panellists could include their own definition of research.

A minimum response rate for Round Two was not set as it was anticipated that the questionnaire rounds would end after Round Two.

A copy of the Round One and Two questionnaires are provided as supplementary material.

### 2.6 Final Steering Committee workshop

In January 2025, the Steering Committee attended an in-person workshop to discuss and agree a final consensus definition.

This approach was taken to allow the wording of the definition to be finalised in a more efficient way than a further Delphi Round and was appropriate because high levels of agreement for the Round Two definition statements were achieved.

Responses to Round Two definition statements were presented for review (see Table 3) and discussion along with panellists’ comments and suggested definition statements. As all the Round Two definition statements had median responses indicating agreement or strong agreement, they were all taken forward for discussion at the workshop.

**Table 3:** Round Two definitions of research with Round Two responses.

Definition Statement	Mode	Median	Mean
Using structured, organised and, where possible reproducible, methods to produce information or knowledge, which may include testing an idea, theory, or new intervention, is research	6	6	5.3
Using structured, organised and where possible, reproducible methods to interpret existing information is research. This may include routinely collected data being used for a new purpose, as well as publicly available data.	6	6	5.3
Producing findings that are generalisable (i.e. are useful beyond the original setting of the work) is research.	5	5	4.7
Research is undertaken to inform decisions about practice and what policies and interventions should be implemented at a local, regional or national level.	5	5	5
Research seeks to help us understand how people are impacted by the context in which they live, with the aim of benefitting communities and reducing inequalities.	5	5	5
Resident/community consultations (e.g. asking members of the public for their views) where this is considered routine practice or business as usual, is not research.	6	4	4
Routine evaluation of local authority services, for internal service monitoring and improvement, is not research.	6	4	3.9

Steering Committee members were provided with written instructions and questions to guide discussions (see supplementary material). They were also reminded that new aspects of a definition (not included in Round Two) could not be introduced at this stage.

### 2.7 Data analysis

For Round One, rates of agreement and disagreement for each definition were calculated as a percentage of the total number of responses to the statement. Only definitions which scored above the threshold of 75% of panellists agreeing were included in Round Two. Panellists’ comments and own definitions of research were analysed by two members of the Steering Committee (LMB, LH) and themes identified. The responses, rates of agreement and disagreement, comments and identified themes were then discussed by the Steering Committee and used to shape the Round Two definitions and inform the presentation and content of the Round Two Questionnaire.

For the Round Two Questionnaire, panellists provided their level of agreement with each definition on a six-point Likert scale (1 = strongly disagree and 6 = strongly agree). The mode, median and mean score for each definition was calculated and the comments made about each definition were analysed and themes identified by two members of the Steering Committee (LMB, LH). These were presented to the Steering Committee along with the range of scores for each definition and number of comments provided about each. Panellists’ own suggested definitions were provided but were not split into themes.

At the final in-person Steering Committee workshop, the Committee worked in two sub-groups. Notes of sub-group and whole committee discussions, and the decisions taken, were documented (see supplementary material).

## 3 Results

The response rate (completed questionnaires) in Round One was 80%.

Of the 24 initial definitions, ten achieved an agreement rate >75%, eleven an agreement rate between 50% and 75% and three an agreement rate <50%. Details of the agreement rates associated with each definition are shown in Table 2. When the ten that achieved >75% agreement were reviewed and it was identified that characteristics were shared between definitions, these were combined, resulting in five definitions for inclusion in Round Two.

The response rate (complete response) for the Round Two Questionnaire was 77%.

For the five definitions of research provided in Round Two, scores ranged from 2 (disagree) to 6 (strongly agree). All definitions had mode and median scores of either 5 or 6 and mean scores between 4.7 and 5.3 (see Table 3).

For the two definitions of what research was not, scores ranged from 1 (strongly disagree) to 6 (strongly agree) with mode scores of 6 and median scores of 4 for both definitions. Mean scores were 3.9 and 4 respectively.

In the workshop the Steering Committee concluded that four of the five Round Two definition statements should feature in the final definition. However, both sub-groups independently concluded that, based on panellist responses and comments, there was insufficient strength of agreement, or support, for ‘generalisability’ to be included in the consensus definition.

It was clear from both the Round One and Round Two responses and comments that the definition of research in a local authority should have two components: the purpose - why the research is done; and process - how the research is done. The Steering Committee agreed that the structure of the final definition should reflect this. The committee decided that the definition should start with purpose before moving on to the process.

It was also agreed that the proposed negative definitions of research (what is not research) did not have sufficient support to feature within the final definition. The record of the discussions and decisions made in the workshop are provided as supplementary material.

The Steering Committee agreed on the following definition statement, which incorporates the four definition statements that had consensus. For an activity to be defined as local authority research, it must meet the criteria for both parts of the definition.

***Part 1***

**Local authority research supports decision making about practice, policies and interventions at a local, regional or national level**

**and/or**

**It helps us understand how people are impacted by the context in which they live, work and go about their daily lives.**

**AND**

***Part 2***

**Research uses structured, organised and reproducible methods to:**

• **produce new information or knowledge, which may include testing an idea, theory or new intervention**

**and/or**

• **provide a new interpretation of existing information. This may include routinely collected data being used for a new purpose, as well as publicly available data.**

## 4 Discussion

This paper describes a study that successfully developed a consensus definition of research in a local authority setting using the Delphi approach. The need for this definition has been noted previously [6]. A consistent, local authority-relevant definition of research is needed to promote a culture of research and evidence informed decision making within local authorities by enabling officers to identify when they are, or could be, involved in research and by making it easier to communicate about research. A consistent definition is also a starting point for determining which activities should be subject to formal research governance or ethical review. However, as outlined by Kolstoe et al. [20], definition alone may not be the best means for determining what activities require external ethical review.

Existing definitions of research utilised by established ethical review systems may not be fit for purpose in a local authority context. Our definition results from a comprehensive consultative process, which included representation from a broad range of local authority officers. This ensures that the definition is owned by those involved in local authority research and can be used by them as a starting point for initiating internal or external ethical and governance review; to identify when resource might be available to support an activity; and to help individuals undertaking local authority research to communicate and disseminate it.

The Delphi method is useful for achieving consensus from a wide range of individuals. The Delphi panel was geographically diverse, from different types of local authority, and from different local authority directorates and roles (including public health consultant, social worker, analyst, enforcement officer). Knowledge and experience of ‘research’ was not a prerequisite although most panellists had an awareness or experience of research either within, or outside, the local authority setting. This ensured that the consensus definition is informed by implicit knowledge of what research looks like and a detailed understanding of a local authority setting.

Although Delphi is an established approach for achieving consensus, there are recognised limitations within the method including potential researcher and panellist bias and questions over how consensus is defined [21].

The consensus definition was finalised by the Steering Committee based on responses to the Delphi and panellist comments. The Delphi panel was not consulted on the structure of the final definition. We decided *a-priori* that the Delphi itself would consist of two rounds and the Steering Committee would agree the specific wording of the definition based on the consensus reached. This is consistent with guidance on the conduct of Delphi studies which suggests that too many rounds could lead to high attrition and false consensus [10]. Our intention is to now test the definition in practice by gathering ongoing feedback.

A potential limitation of this approach is in the recruitment and retention of a panel of appropriate experts. Clear information about expectations and timelines for participation in the study was provided to panellists to mitigate this. The success of this strategy and the enthusiasm of the panel about the topic is reflected in the high response rates for both Delphi rounds.

Attempts were made to ensure a diverse panel that reflected a range of local authority directorates and job roles however we note that the majority of the panel (51%), and several members of the Steering Committee, were employed in Public Health teams. A large proportion of panellists (48%) were recruited from local authorities with NIHR Health Determinants Research Collaboration funding. We acknowledge that a culture of research informing decision making is more established in Public Health compared to other local authority departments [5]. This could be a limitation for applicability of the definition to other local authority directorates. Local authorities are diverse in their structures, organisational cultures and research systems [5]. Further work is now needed to test the appropriateness of the definition across this diversity. This is something that we are exploring as we disseminate the definition and seek feedback on its usefulness.

The initial driver for this project was the need to define research so that a consistent approach to research governance and ethics processes within local authorities could be taken [6]. During this work, it became clear that an important function of the definition would be to help local authority officers understand what research is and why it is important in developing the evidence base to inform decision making. The need for ethical or governance review was not a factor that panellists were asked to consider for potential definitions, meaning the final agreed definition is inclusive, covering a wide spectrum of activities. This is consistent with other work published recently around there being a difference between ‘research’ and ‘research that requires ethical review’ [20]. We acknowledge that many activities within a local authority setting meeting our consensus definition will not require independent ethical review or formal ‘research governance’ approval processes.

Next steps for this work are for the definition to be circulated as widely as possible to local authority officers so that they can judge its usefulness, both in terms of how local authorities think about their own work, and in terms of communicating evidence generation practices to external stakeholders. We are seeking feedback on its use so it can be refined in response. A subsequent piece of work looking at the relevance of this definition to wider contexts (e.g. VCSE sector) may also be appropriate. Guidance to accompany the definition including a decision tool has been developed (see supplementary material) and feedback on this resource and how the definition works in practice is actively being collected.

In conclusion, we have developed a collective consensus definition of what research in a local authority setting is. This has utility in helping local authority officers to recognise and communicate about research and its importance in a local authority setting and to provide a starting point for considering the need for formal research governance or independent ethical review.

It is important in terms of developing a research culture, to ensure decision making in local authorities is evidence informed, and that local authority officers recognise research as something that they can and are doing [7] rather than seeing it as something that is done “over there” in universities. The development of the broad definition of research in a local authority developed in this project with partners working in the system will help achieve this.

## Ethical statement

The study received a favourable ethics opinion from Newcastle University Faculty of Medical Sciences (FMS) Research Ethics Committee (2804/48670).

## CRediT authorship contribution statement

Conceptualization and Methodology: All authors. Investigation: LH and LMB. Formal analysis: LH and LMB. Project administration: LH and LMB. Writing- original draft: LMB and LH. Writing-review and editing: All authors.

## Funding

This study was funded by the 10.13039/501100000272NIHR Research Support Service Specialist Centre for Public Health.

The views and opinions expressed in this paper are those of the author(s) and not necessarily those of the NIHR.

## Declaration of competing interest

The authors declare the following financial interests/personal relationships which may be considered as potential competing interests:

ADMB is part-funded by NIHR in his role as Public Health Research Programme Director.
