# Supplementary material for: A local-authority specific definition of research: Results from a Delphi study
Source: Public Health Pract (Oxf). 2026 Mar 4;11:100765. doi: 10.1016/j.puhip.2026.100765 (PMC12996929; doi:10.1016/j.puhip.2026.100765)
Supplement: Multimedia Component 1 [file mmc1.docx]

**Definition of Applied Local Government and Community-based Research**

**Summary Notes from Steering Committee Workshop**

**Monday 13 January 2025**

| **Attendees:** | REDACTED |
| --- | --- |
| **Observers:** | REDACTED |
| **Apologies:** | REDACTED |

**Welcome and introductions provided**

**Summary of the project so far and Round Two responses given**

Discussed that the high level of agreement on the Round Two definitions was useful.

**Workshop discussions**

- Split into two groups and discussed the five Round Two definitions of research and also the two statements of what research is not.

**Whole group discussion**

Table 2 feedback:

- **Statement 3-** Agreed as group to remove this definition- rationale is lowest level of agreement from Round Two/comments. Not all activity that would be considered research is necessarily generalisable beyond the setting in which it was done.
- **Statements 1 and 2**- Leave unchanged.
- Had most discussion as a group around **statement 4**. Felt that the ‘supports practice’ might be better as an alternative to ‘informs practice’, Questions around whether some research will directly inform policy- might be further away from this (e.g. pilot work) and so might not directly ‘inform’, although could be considered on a pathway to informing/influencing policy.
- **Statement 5-** Leave unchanged. Good that references to inequalities have gone.
- Some discussion around whether research is *EITHER* 1 *OR* 2 *AND* 4 *OR* 5- e.g. does not need to be 1 *AND* 2 *AND* 4 *AND* 5.
- Struggled with **statements 6 and 7** regarding what research is not. Tending towards not including within a definition explicitly but might be useful to have as a filter in a decision tool and then also with some accompanying text outlining that even ‘non-research’ activities should be managed and conducted in a robust and ethical way.
- Potentially more work needed to refine wording around what research is not.

Table 1 feedback:

- **Statement 3**- Also agreed to remove for the same rationale as Table 2.
- **Statements 1 and 2**- Have suggested blending these into a single definition and tweaking slightly.

*‘Research is using structured, organised and reproducible methods to either produce new information and knowledge or provide a new interpretation and understanding of existing information. Research tests an idea, theory or new intervention. It may use routinely collected data for a new purpose including publicly available data’*

- See **statements 4 and 5** as being useful as an introduction to the definition and thought it might make sense to have these before statements 1 and 2.
- Tweaks made to **Statement 4** – include local authority research- to make it explicit to local government. Have added ‘making’ to specify ‘decision-making’ rather than just decisions. Have also removed implementation in acknowledgement that research might result in services being cut back as well as developed.
- **Statement 5-** have removed ‘seeks to’ to make this more definite.
- Had difficulty with **statements 6 and 7-** as hard to pin down what research isn’t. Tending toward staying away from saying what research isn’t which is a negative. Feel that can maybe address this in some accompanying text to the definition- e.g. statement to say that it can be hard to define what research isn’t and that this is an issue faced by many. Can maybe direct to sources of information/advice on this (e.g. HDRC, LARP, SCPH).

**Further whole group discussion on whether to include definitions of what research IS NOT**

- One question to consider with this is the point of view of research participants and when they would need to know whether the activity is research or not and if it would feel different to them if they were involved in research (as opposed to being part of a consultation, for example).
- Noted that in the Delphi surveys we only tested two potential definitions of what research is not and these are not comprehensive. There are many more examples have not been tested. We have probably not captured enough feedback on this to include.

**Final decision on consensus definition**

- Discussion on whether method development should be incorporated along with statements 1 and 2. Agreed that it is already implied in statements 1 and 2 but cannot be explicitly referenced as views on this were not sought in Rounds One and Two of the Delphi.
- Agreed that statements 4 and 5 (as tweaked) will form the beginning of the definition.
- Agreed that statements 1 and 2 will remain separate but will have a common stem to avoid repetition and then be presented as bullet points e.g. using structured, organised and reproducible methods to:

Produce new information…

Provide a new interpretation…

- Agreed that statements 4 and 5 will be separated by and/or and so will statements 1 and 2. However 4/5 and 1/2 as concepts will be separated by AND (e.g. to meet the definition will need to meet at least one of 4 or 5 **and** at least one of 1 and 2.
- Agreed as a group that statements of what research is not should not be included in the definition but that reference should be made to this in accompanying text. Might also be useful to recognise that some work uses research skills but may not require formal research governance or ethical review.

**ACTION: Final consensus definition will be drafted to reflect the above decisions and circulated to the steering group for review. (Owner: LB)**

- Agreed that ultimately it will be a local decision as to what will be captured within a local authority’s research governance process and what activities may be exempt from this or will require a different review pathway.
- Agreed that a decision tool can be developed based on this work which is helpful in outlining the territory but without being too directive/prescriptive. Will need to include wording which makes it clear that this is an advisory tool to assist in decision making, but that local research governance and ethics processes should still be followed.
- Also worth including some preamble to explain that consultation activities which may not be classed as research may still have ethical issues and governance requirements which needs to be reviewed/addressed.
- Agreed that this tool should be different to the HRA type tool and should acknowledge that there are grey areas and that this is where advice, support and local decision-making will be key.
- Agreed that some examples should be provided to help with the definition. Providing actual examples from NIHR funded studies and also practitioner fellowship projects would be helpful. Can also include example relating to methods development.

**ACTION: Examples will be ‘sourced’ and circulated to the group for review. (Owner: LB)**

**Discussion around decision tool**

The remaining time at the workshop was spent discussing a decision tool based on the definition.

- Discussed starting with information on why the tool is needed/when it should be used.

**Why might you want to use this tool?**

- - To establish if the activity you plan to do requires formal ethics/governance approval (*do we want to say from LA - or will that muddy waters more re service evaluations etc. that LAs already have processes for approving?)*
  - To help to guide the activities that HDRC/LA Research Support Team support
  - If you are unsure if what you are doing/planning to do is research and want some guidance
  - To explore if your idea would allow you to access a resource - i.e. is it eligible for research funding/support from SCPH; would it be attractive to an academic partner
  - *Post meeting note - do we want here (or somewhere) to say something about if intend to publish findings in academic journal - i.e. would need to have gone through ethical review*
- Important to have a statement acknowledging that LAs have their own governance procedures and this doesn't replace these; it's intended as a guide for individuals/LAs who find it helpful to help them to think through if the work they plan to do is research.
- Also talked about including as part of this "package" (that will accompany our agreed definition of research in an LA context) signposting to training in ethical research practice and sources of support (e.g. SCPH, HDRCs etc.)
- Agreed that it will be important to have the decision tree visible all at once (rather than revealing next steps based on responses) this will help in terms of providing whole picture.
- Might be helpful to separate out certain components especially from the process side e.g. structured, organised, reproducible.
- Agreed that where the flow ends in a ‘No’ or ‘Not research’ there is a statement to advise that other processes may still need to followed and guidance on next steps/sources of support (which will also be provided for when conclusion is that the activity is research)

**ACTION: To work on draft flow-chart and accompanying preamble to then circulate to the Steering Committee for review. (Owner: LH/LB)**

**ACTION: Follow-up meeting of the Steering Group to discuss examples and next steps to be arranged as soon as possible (Feb/early March). It would be great to be in-person but recognised that due to availability will need to be virtual. (Owner: LB)**

**Consensus definition:**

Purpose (the ‘why’)

Local authority research supports decision making about practice, policies and interventions at a local, regional or national level

*and/or*

It helps us understand how people are impacted by the context in which they live, work and go about their daily lives.

Process (the how)

Research uses structured, organised and reproducible methods to:

- produce new information or knowledge, which may include testing an idea, theory or new intervention

*and/or*

- provide a new interpretation of existing information. This may include routinely collected data being used for a new purpose, as well as publicly available data.
