# Supplementary material for: A local-authority specific definition of research: Results from a Delphi study
Source: Public Health Pract (Oxf). 2026 Mar 4;11:100765. doi: 10.1016/j.puhip.2026.100765 (PMC12996929; doi:10.1016/j.puhip.2026.100765)
Supplement: Multimedia Component 2 [file mmc2.pdf]

Information Sheet

Definition of Research in Local Authorities Project

Invitation and Brief Summary

You are being invited to take part in a project which aims to achieve a consensus definition of research in a Local Authority setting.

This will involve completing two online questionnaires approximately six weeks apart.

Before you decide whether or not you wish to take part it is important that you understand why the project is being done and what taking part will involve.

Please read this information carefully. If you do decide to take part, you will be asked to provide consent. However, you are free to withdraw at any time, and without giving any reason.

What is the purpose of the project?

There is a lack of consistency both between and within Local Authorities (LAs) on what they define as research, for example in the context of the wider determinants of health.

In some LAs, research is defined as projects that align with a definition of research provided in the UK Governance Framework for Health and Social Care Research whereas others have a broader definition that includes a continuum of research-like activities including resident engagement and evaluation.

The lack of a common definition of research is hindering activity to establish robust research infrastructure, ethics and governance processes and a research active culture in LAs.

This project aims to develop agreement amongst a range of individuals from LAs in the UK on how research, in areas such as the wider determinants of health, is defined in Local Authorities.

Why have I been invited to take part?

We are inviting staff based in Local Authorities in the UK to take part in this project.

We are interested in hearing from individuals in a range of roles and departments within local government.

What will taking part involve?

Taking part in the project will involve completing two rounds of online questionnaires.

In the first round, you will be asked to review statements about a potential definition of research in a LA context and agree or disagree with them.

For each statement there is the opportunity to provide free text or comments to explain the reasoning for your selection. Additional comments would be welcomed by the research team to help us to understand the choices you make. At the end of the questionnaire, you will also be able to suggest additional definitions that you feel have not been included.

In the second round you will receive an updated list of definitions based on the statements that had the highest level of agreement in the first round and taking into account the

comments and suggestions received. You will be asked to respond to these to confirm your agreement and provide any relevant comments.

Following completion of Round Two, the research team and project Steering Committee will review the responses to determine which statements have the highest level of agreement. From these we hope to create a consensus definition of research.

Each questionnaire round will take approximately 20-30 minutes to complete and Round Two will happen approximately six weeks after the end of Round One.

Each questionnaire round will be open for three weeks and we will send email reminders out approximately two weeks into each round.

If, after reading this information, you are interested in taking part, we will ask you to confirm your consent and provide some details about your job role, the type of LA you are based in and your geographical region.

We will also ask you to provide your email address so that we can send you the questionnaires and project updates by email.

We anticipate including 30-40 respondents (panellists) in our project and want to get good variation in terms of job role, type of LA and geographical location.

We hope that we will be able to involve every person who expresses interest in the project however we cannot guarantee this. If you are not selected to proceed to the questionnaire rounds, we will let you know and will also ensure that you are updated about the project and its results.

We are planning to send out the first questionnaires in September 2024 and the second questionnaires in late November/early December 2024.

What information will be collected and who will have access to the information collected?

As part of this project, the only identifiable information we will collect about you will be your first name and email address which we will use to send out the questionnaires and to contact you about the project.

We will also ask you for details about your job role and experience in this role and about the type of Local Authority in

which you are based. This will include asking for the region in which you are based.

All other information you will be asked to provide will relate to your opinion on the research definition statements.

Data will be stored securely within the questionnaire software and at Newcastle University. Only authorised members of the research team at Newcastle University will have access to your email address and questionnaire responses.

Data will be summarised and shared with the project steering committee; however, these data will be de-identified. No one within, or outside your organisation, other than the research team, will know that you have taken part in this project.

To make data collection easier we may offer a 'voice to text' facility which allows you to record an audio response to a question. Where this is the case, the audio will immediately be transcribed into text and we will not be storing any recordings of your voice.

The online survey software we use will be GDPR compliant and secure and only authorised members of the research team will have access to questionnaire responses.

Once we have finished the project, we will keep some of the data (for example the responses to questions and details about your role and LA) so we can check the results.

We will write our reports in a way that no-one can work out that you took part. However, anonymised free text comments may be used in the write-up of this work and in peer-reviewed publications and presentations.

The data may be de-identified and may be shared with other researchers in the UK (and potentially overseas). It may also be made available as “open data” through a research data repository. This means the de-identified data will be publicly available and may be used for purposes not related to this project.

Who is the sponsor and data controller for this research?

Newcastle University is the sponsor for this project. Newcastle University will be using information from you in order to undertake this project and will act as the data controller. This means that Newcastle University is responsible for looking after your information and using it properly.

The lawful basis for carrying out this study under GDPR is Task in the Public Interest, (Article 6,1e) as research is cited as part of the University’s duties.

Identifiable data from this study (email address) will be kept by Newcastle University for up to 12 months after the project has ended. De-identified data (e.g. where you are not directly

identifiable) and summaries of the project results will be held for longer (indefinitely).

Individuals at Newcastle University may look at your research data to check the accuracy of the research. The only individuals at Newcastle University who will have access to information that identifies you will be the research team or individuals auditing the project or the data collection process.

Your rights to access, change or move your information are limited, as Newcastle University need to manage your information in specific ways in order for the research to be reliable and accurate. If you withdraw from the project, Newcastle University will keep the information about you that has already been obtained. To safeguard your rights, the minimum personally-identifiable information will be used.

You can find out more about how Newcastle University uses your information at **ncl.ac.uk/data-protection/** and/or by contacting their Data Protection Officer: **rec-man@newcastle.ac.uk**

What are the possible benefits or disadvantages of taking part?

There will be no direct benefits to you personally from taking part in this project however we hope that if we can achieve consensus on a definition of research in a LA context it will help LAs improve and streamline their research processes.

A possible disadvantage is that you will have to spend some time completing the questionnaires. Each questionnaire will take approximately 20-30 minutes to complete, however you will be able to save your responses and return to them later if needed.

What will happen at the end of the project?

Once you have completed both rounds of questionnaires, your participation in the project will end. Once the overall project has been completed, the research team will contact you to let you know the results and any consensus definition of research that has been agreed.

Who is funding this project?

This project is funded by the NIHR Research Support Service Specialist Centre for Public Health.

Has this project received ethical approval?

This project has received ethical approval from the Newcastle University Ethics Committee.

Who should I contact for further information relating to the project?

Principal Investigator Louise Hayes,
louise.hayes@newcastle.ac.uk
Project Contact: Laura Brown,
laura.brown8@newcastle.ac.uk

If you wish to discuss the project or have any concerns, you can contact a member of the project team on the details above.

If you wish to raise a complaint on how your personal data is handled, you can contact the Newcastle University Data Protection Officer Data Protection Officer who will investigate the matter: **rec-man@newcastle.ac.uk**
If you are not satisfied with their response you can complain to the Information Commissioner's Office (ICO): **www.ico.org.uk**

Please continue onto the next page to provide consent.

Consent

Definition of Research in Local Authorities Project

Consent Statements

- I have read and understood the information about this project.

- I understand that expressing interest and completing the initial following questions does not guarantee that I will be selected for the questionnaire rounds of the project.

- I understand what my participation will involve and that I am free to withdraw at any time without giving any reason.

- I understand that if I decide to withdraw, any data I have provided up to that point will be kept and used.

- I agree to the storage and use of my personal information, including email address, for the purposes of this project, as described in the information sheet.

Please indicate whether you consent

- ◯ I consent to take part- continue with the questionnaire
- ◯ I do not consent to take part

Panellist Information

Please provide your first name (we will use this to address emails to you)

Please provide your email address (we will use this to send out the Definition of Research questionnaires)

What is your job title?

Please indicate your type of role

- ◯ Director/Head of a Service or equivalent
- ◯ Senior/Top Level Management
- ◯ Mid-Level Management
- ◯ Entry-Level Management
- ◯ Elected member
- ◯ Other

Please provide details of your professional background if applicable (e.g. planner, scientific, registered healthcare professional)

Please briefly describe your role, for example, if you are an elected member we would be interested to know if you are a

portfolio holder or Committee Chair

(you can speak or type your response)

Record Speech New Record Speech Continue

Please indicate your directorate or department type

- Public Health
- Housing
- Social Care
- Corporate/Central
- Communities
- Planning/Regulatory
- Other

If other, please specify your department name or type

How long have you worked, or been based in a Local Authority?

- Less than 1 year
- 1-5 years
- 5-10 years
- 10-15 years
- 15-20 years

- More than 20 years

What type of LA are you based in

- Tier 1 (Metropolitan, County, Unitary or London Borough)
- Tier 2 (District)

In what geographical region is your LA?

- East of England
- East Midlands
- London
- North East
- North West
- Northern Ireland
- Scotland
- South East
- South West
- Wales
- West Midlands
- Yorkshire & the Humber

Local Authority Research Stages Model

Research Launch (R-LAUNCH)	Research Build (R-BUILD)	Research Enhance (R-ENHANCE)
No (or very limited) research experience as an organisation.	Some research experience as an organisation.	Established research experience as an organisation.
Research processes or infrastructure not yet fully considered.	Research processes and infrastructure being considered or developed.	Some research processes and infrastructure in place.
Not yet explored external research funding opportunities.	Beginning to explore external research funding opportunities.	External research funding opportunities secured.

Referring to the above model of research stages, at what stage would you consider your LA to be?

If you need the above information in an accessible format please contact laura.brown8@ncl.ac.uk

- ◯ R-LAUNCH
- ◯ R-BUILD
- ◯ R-ENHANCE

◯ Unsure

Does your Local Authority have Health Determinant Research Collaboration (HDRC) status?

◯ Yes
◯ No
◯ Unsure

Please select the option which best describes your personal 'research' experience.
(By 'linked to research' we mean any role which involves doing or supporting research or researchers)

◯ Currently working in role within a LA which is linked to research
◯ Previously worked in a role within a LA which is linked to research but now in a different non-research role
◯ No experience of research within a LA, but previous experience linked to research in other organisations
◯ Currently in a role within a LA requiring an awareness of 'research' but not directly involved
◯ Currently in a role with no links to research but have had previous experience/links
◯ Currently in a role with no links to research and no previous experience/links

Block 3

Thank you for expressing an interest in the project and completing the initial questions.

When you continue onto the next page, your responses will be saved and sent to the research team. A copy of your responses will also be emailed to you.

Over the next few weeks we will review all responses and select respondents for the two research definition survey rounds.

We will contact you by email (after 06 September 2024) to let you know whether or not you have been selected for the survey rounds and to provide you with information about the next steps.

In the meantime if you have any questions or would like to withdraw from the project please contact the research team:
Louise Hayes, louise.hayes@newcastle.ac.uk
Laura Brown, laura.brown8@newcastle.ac.uk

Powered by Qualtrics
