# Supplementary material for: A local-authority specific definition of research: Results from a Delphi study
Source: Public Health Pract (Oxf). 2026 Mar 4;11:100765. doi: 10.1016/j.puhip.2026.100765 (PMC12996929; doi:10.1016/j.puhip.2026.100765)
Supplement: Multimedia Component 3 [file mmc3.pdf]

Introduction

Research Definition Consensus Project - Round 1 Survey

Thank you for agreeing to be part of this project which aims to find a consensus definition for research in a Local Authority (LA) setting.

By continuing with the survey you are confirming your continued consent to take part.

In the following survey, we will provide you with a set of potential definitions of research. For each definition, please select if you agree or disagree with it. You do not need to feel that a particular definition applies to all research to agree with it. It might be that a particular definition covers, or partly covers, one aspect or type of research.

You can therefore agree or disagree with as many of the statements as you like.

You will also be given the option to add some comments against each definition, for example to explain why you have agreed or disagreed, or to point out any parts of the definition you would change. We would really welcome comments, especially where you feel that part of a single definition may be more relevant than its other parts.

You can type these additional comments, or can speak them by pressing 'Record Speech Now' and the survey will transcribe your speech for you. You can stop and then continue speaking using the 'Record Speech Continue' button.

The survey link you used to access this survey is unique to you. If you need to leave the survey before completing it, your progress will automatically be saved and you will be able to return to complete your responses (if using the same browser and machine) by clicking on your original link.

Email Address reconfirmation

Please enter your email address (so that we can send you a copy of your completed questionnaire response)

Statement 1

Research includes any activity that **assesses a novel intervention by randomly allocating participants to receive the intervention or an alternative.**

- ○ Agree
- ○ Disagree

Comments

Record Speech New | Record Speech Continue

Statement 2

Research includes any activity that **involves a systematic investigation designed to develop or contribute to generalisable knowledge.**

- ○ Agree
- ○ Disagree

Comments

Record Speech New | Record Speech Continue

Statement 3

Research includes any activity that **addresses a question with scientifically sound (and reproducible) methods and has clearly defined aims and objectives.**

- ○ Agree
- ○ Disagree

Comments

Record Speech New | Record Speech Continue

Statement 4

Research includes any activity that **involves collecting additional data to that collected routinely.**

○ Agree
○ Disagree

Comments

Record Speech New | Record Speech Continue

Statement 5

Research includes any activity that **uses existing, routinely collected data (secondary data) in a new way to provide insights and guide new activity.**

○ Agree
○ Disagree

Comments

Record Speech New | Record Speech Continue

Statement 6

Research includes any activity that **involves the systematic collection, analysis, and interpretation of data relating to an area of focus (e.g. the wider determinants of health).**

- ○ Agree
- ○ Disagree

Comments

Record Speech New | Record Speech Continue

Statement 7

Research includes any activity that **involves evaluating existing, already in use, interventions or services with the aim of reviewing, improving or developing them (including judging how well a service is performing, possibly against a predetermined standard).**

- ○ Agree
- ○ Disagree

Comments

Record Speech New Record Speech Continue

Statement 8

Research includes any activity that **includes evidence syntheses activities (bringing together data from multiple sources to provide a summary of existing knowledge) e.g. systematic review, meta-analysis.**

- ○ Agree
- ○ Disagree

Comments

Record Speech New Record Speech Continue

Statement 9

Research includes any activity that **may be co-designed and co-produced with the local community/service users.**

- ◯ Agree
- ◯ Disagree

Comments

Record Speech New | Record Speech Continue

Statement 10

Research includes any activity that **involves administering a questionnaire to, or using other methods (e.g. focus groups) to gather the views of, the public, service users or staff on a particular subject, service, or issue.**

- ◯ Agree
- ◯ Disagree

Comments

Record Speech New | Record Speech Continue

Statement 11

Research includes any activity that **involves collecting information on the views and experiences of a defined population e.g. residents of a specific town, city or region.**

- ◯ Agree
- ◯ Disagree

Comments

Record Speech New | Record Speech Continue

Statement 12

Research includes any activity that **has a primary aim of producing or contributing to generalisable or transferable new knowledge to answer or refine relevant questions using scientifically sound methods.**

○ Agree
○ Disagree

Comments

Record Speech New | Record Speech Continue

Statement 13

Research includes any activity that **aims to ask a question which has not yet been answered (i.e. to tell us something new) and which may be used as the basis for decision making.**

○ Agree
○ Disagree

Comments

Record Speech New | Record Speech Continue

Statement 14

Research includes any activity that **focuses on understanding population behaviour and issues, considering factors (e.g. socioeconomic, cultural, environmental) of influence to improve health and wellbeing.**

- ◯ Agree
- ◯ Disagree

Comments

Record Speech New | Record Speech Continue

Statement 15

Research includes any activity that **aims to produce findings to inform policy and what interventions should be invested in, for example to improve population health and wellbeing and reduce health inequalities.**

- ◯ Agree
- ◯ Disagree

Comments

Record Speech New Record Speech Continue

Statement 16

Research includes any activity that **has the primary intention of preventing disease or injury or improving an existing programme or service.**

- ◯ Agree
- ◯ Disagree

Comments

Record Speech New Record Speech Continue

Statement 17

Research includes any activity that **will potentially save LAs money, or allow LA budgets to be more accurately**

targeted, via new approaches or insights which will allow efficiency savings to be made.

- ◯ Agree
- ◯ Disagree

Comments

Record Speech New | Record Speech Continue

Statement 18

Research includes any activity that **generates information for internal use only.**

- ◯ Agree
- ◯ Disagree

Comments

Record Speech New | Record Speech Continue

Statement 19

Research includes any activity that **aims to generate a publishable output (e.g. academic journal article).**

○ Agree
○ Disagree

Comments

Record Speech New | Record Speech Continue

Statement 20

Research includes any activity that **aims to produce an output that could be of use or interest beyond the immediate service/area where the activity is taking place.**

○ Agree
○ Disagree

Comments

Record Speech New | Record Speech Continue

Statement 21

Research includes any activity that **intends to identify ways to improve the wellbeing, health or efficiency of people employed by, or working with, the LA.**

- ○ Agree
- ○ Disagree

Comments

Record Speech New | Record Speech Continue

Statement 22

Research includes any activity that **involves collecting data with, or on behalf of, an academic institution (e.g. a University) including for the purpose of obtaining a formal qualification.**

- ○ Agree

◯ Disagree

Comments

Record Speech New | Record Speech Continue

Statement 23

Research includes any activity that **is externally funded via a research funding body, charity research funding stream or commercial entity.**

◯ Agree
◯ Disagree

Comments

Record Speech New | Record Speech Continue

Statement 24

Research includes any activity that **falls outside of, or goes beyond, routine practice/business as usual and that may represent a risk to individuals, or the organisation, if not managed appropriately.**

- ◯ Agree
- ◯ Disagree

Comments

Record Speech New | Record Speech Continue

Final Comments

Please feel to suggest your own definition of research or provide any additional comments

Record Speech New | Record Speech Continue

Powered by Qualtrics
