# Supplementary material for: A local-authority specific definition of research: Results from a Delphi study
Source: Public Health Pract (Oxf). 2026 Mar 4;11:100765. doi: 10.1016/j.puhip.2026.100765 (PMC12996929; doi:10.1016/j.puhip.2026.100765)
Supplement: Multimedia Component 4 [file mmc4.pdf]

## Introduction

## Research Definition Consensus Project - Round Two Survey

Thank you for being part of this project. We really appreciate your input.

This survey (Round Two) is the second stage of the project.

Before the survey starts, we have included a brief reminder of this project's purpose and context.

You may find this helpful as you think about the Round Two definitions.

## Background and context

There are inconsistencies in how local authorities (LAs)

currently define research within a local authority setting.

Some use the UK Policy Framework for Health and Social Care Research definition. Other LAs have a broader definition, incorporating activities like resident engagement and evaluation.

Without a consistent and easily understood definition of research in a local authority, it is difficult to talk about research in LAs.

This makes it hard for LAs to recognise, develop, promote, and use research and evidence. Determining when research governance and ethical review processes may be needed is also then a challenge.

This project aims to develop agreement on how research is defined in LAs.

There are differences between how a research activity is done (process) and the purpose for which it is done. Both these factors may be important for a research definition. We have reflected this in the definitions we are asking you to evaluate in this Round Two of the project.

**Instructions for Round Two**

In Round One we provided initial definitions of research and asked you to agree or disagree with each. Comments and suggested definitions were also invited.

Based on the Round One responses, we have created updated definitions. These incorporate the definitions from Round One

that most (over 75%) panellists agreed with. They also take into account comments and suggested definitions.

In this round, instead of simply indicating that you ‘agree’ or ‘disagree’ with a definition, you are being asked to indicate how strongly you disagree or agree with a definition on a scale from 1 to 6.
**1** indicates you very **strongly disagree** the statement ***is*** research, **6** means you very **strongly agree** the statement ***is*** research.

When deciding the extent to which you agree, we would like you to think about whether you consider the activities described in the definition to be research or not. You do not need to think that the draft definition covers the entirety of what research is in order to agree with it. You just need to focus on if you think what is described would 'count' as being a **component** of research.

Comments on the updated definitions and examples are helpful, especially where you partly agree with a definition, or where your agreement or disagreement is not strong. These will help us identify which parts of the definitions and which ideas are most relevant.

You can type comments, or can speak them by pressing 'Record Speech Now' and the survey will transcribe your speech for you. You can stop and then continue speaking using the 'Record Speech Continue' button.

After you have responded to the updated definitions and examples, we then provide some potential definitions/explanations of what research **is not.**

We will ask you whether you agree or disagree with these using the same scale and comments.
**1** indicates you very **strongly disagree** the statement ***is not*** research, **6** means you very **strongly agree** the statement ***is not*** research.

At the very end of the survey, we will also ask you for some brief feedback about being part of this project.

The survey link you used to access this survey is unique to you. If you need to leave the survey before completing it, your progress will automatically be saved, and you will be able to return to complete your responses (if using the same browser and machine) by clicking on your original link.

If you have any problems with the survey or have any questions, please contact nihr.rss.publichealth@newcastle.ac.uk or laura.brown8@newcastle.ac.uk

By continuing with the Round Two survey, you are confirming your continued consent to take part.

## Email Address reconfirmation

(Optional) please enter your email address (so that we can send you a copy of your completed questionnaire response)

## Statement 1

The updated statement you will see below is based on a number of original definitions that had a high level of agreement. These definitions had a similar theme around producing knowledge using structured methodology:

| Original Definition Statement | % Agreement |
|---|---|
| Q3. Research includes any activity that addresses a question with scientifically sound (and reproducible) methods and has clearly defined aims and objectives. | 96% |
| Q12. Research includes any activity that has a primary aim of producing or contributing to generalisable or transferable new knowledge to answer or refine relevant questions using scientifically sound methods. | 96% |
| Q2. Research includes any activity that involves a systematic investigation designed to develop or contribute to generalisable knowledge. | 90% |
| Q1. Research includes any activity that assesses a novel intervention by randomly allocating participants to receive the intervention or an alternative. | 82% |

**Updated Definition:**

**Using structured, organised and, where possible reproducible, methods to produce information or knowledge, which may include testing an idea, theory, or new intervention, is research.**

***For example:***

***Conducting a resident survey to assess what different factors might impact wellbeing in the local population.***

***Conducting a focus group with residents living close to a cycleway to explore the barriers and facilitators to using it. Focus group notes or transcripts are analysed in a systematic way using a recognised approach to analysis.***

On the following scale (1 being strongly disagree and 6 being strongly agree) please select the extent to which you agree with the above definition.

○ 1 ○ 2 ○ 3 ○ 4 ○ 5 ○ 6

Comments

Record Speech New Record Speech Continue

The updated statement you will see below is based on a number of original definitions that had a high level of agreement. These definitions had a similar theme around use of data:

| Original Definition Statement | % Agreement |
|---|---|
| Q8. Research includes any activity that includes evidence syntheses activities (bringing together data from multiple sources to provide a summary of existing knowledge) e.g. systematic review, meta-analysis. | 96% |
| Q14. Research includes any activity that focuses on understanding population behaviour and issues, considering factors (e.g. socioeconomic, cultural, environmental) of influence to improve health and wellbeing. | 88% |
| Q6. Research includes any activity that involves the systematic collection, analysis, and interpretation of data relating to an area of focus (e.g. the wider determinants of health). | 84% |
| Q5. Research includes any activity that uses existing, routinely collected data (secondary data) in a new way to provide insights and guide new activity. | 79% |

**Updated Definition:**

**Using structured, organised and where possible, reproducible methods to interpret existing information is**

**research. This may include routinely collected data being used for a new purpose, as well as publicly available data.**

***For example:***

***Analysing social care data to understand potential reasons for higher service use.***

***Linking existing data sources to be able to do new statistical analysis on a local population. .***

On the following scale (1 being strongly disagree and 6 being strongly agree) please select the extent to which you agree with the above definition.

○ 1 ○ 2 ○ 3 ○ 4 ○ 5 ○ 6

Comments

Record Speech New | Record Speech Continue

The updated statement you will see below is based on a number of original definitions that had a high level of agreement. These definitions had a similar theme around producing generalisable knowledge:

| Original Definition Statement | % Agreement |
|---|---|
| Q12. Research includes any activity that has a primary aim of producing or contributing to generalisable or transferable new knowledge to answer or refine relevant questions using scientifically sound methods. | 96% |
| Q2. Research includes any activity that involves a systematic investigation designed to develop or contribute to generalisable knowledge. | 90% |

## Updated Definition:

## Producing findings that are generalisable (i.e. are useful beyond the original setting of the work) is research.

***For example:***

***Quantifying the impact of locally banning advertising of unhealthy food on public transport which could be used to support similar approaches elsewhere in the country.***

***Linking with a local service that supports unemployed young people to identify effective types of support. Situating findings in broader literature***

***would increase confidence that they will work across services and settings.***

On the following scale (1 being strongly disagree and 6 being strongly agree) please select the extent to which you agree with the above definition.

1 2 3 4 5 6

Comments

Record Speech New Record Speech Continue

## Statement 4

The updated definition you will see below is based on a number of initial definitions that had a high level of agreement. These definitions had a similar theme around the purpose of research as a way to inform decision making:

| **Original Definition Statement** | **% Agreement** |
|---|---|
| **Q15. Research includes any activity that aims to produce findings to inform policy and what interventions should be invested in, for example to improve population health and wellbeing and reduce health inequalities.** | **92%** |

| | |
|---|---|
| **Q13. Research includes any activity that aims to ask a question which has not yet been answered (i.e. to tell us something new) and which may be used as the basis for decision making.** | **83%** |
| **Q5. Research includes any activity that uses existing, routinely collected data (secondary data) in a new way to provide insights and guide new activity.** | **79%** |

## Updated Definition:

## Research is undertaken to inform decisions about practice and what policies and interventions should be implemented at a local, regional or national level.

***For example:***

***Evaluating the impact of a new healthy workplace initiative, on staff health, wellbeing and absenteeism.***

***A project to explore the impact of introducing AI into social care practice.***

On the following scale (1 being strongly disagree and 6 being strongly agree) please select the extent to which you agree with the above definition.

○ 1 ○ 2 ○ 3 ○ 4 ○ 5 ○ 6

Comments

Record Speech New | Record Speech Continue

The updated definition you will see below is based on an initial definition that had a high level of agreement. This definition looked at the purpose of research as a way producing benefit and reducing inequalities:

| Original Definition Statement | % Agreement |
|---|---|
| Q15. Research includes any activity that aims to produce findings to inform policy and what interventions should be invested in, for example to improve population health and wellbeing and reduce health inequalities. | 92% |

**Updated Definition:**

**Research seeks to help us understand how people are impacted by the context in which they live, with the aim of benefitting communities and reducing inequalities.**

***For example:***

***Using local authority and NHS data, alongside interviews and surveys, to understand the contribution of falls to social and health care demand among different communities.***

***Collecting and analysing information from a range of people to understand***

***why residents do and don’t use local parks and recreation areas to inform innovative improvement of these facilities.***

On the following scale (1 being strongly disagree and 6 being strongly agree) please select the extent to which you agree with the above definition.

| 1 | 2 | 3 | 4 | 5 | 6 |
|---|---|---|---|---|---|
| ○ | ○ | ○ | ○ | ○ | ○ |

Comments

Record Speech New Record Speech Continue

**What research is NOT**

The following questions ask for your opinion on a description of what research **is not**. (It is recognised that the activities described below may involve similar methods, skills and governance/ethical consideration to research):

**Resident/community consultations (e.g. asking members of the public for their views) where this is considered routine practice**

**or business as usual, is not research.**

***For example:***

***Holding a discussion within a regular mental health support group, to gather views on their satisfaction with local services.***

***Consulting with residents around the potential closure of a local high school.***

On the following scale (1 being strongly disagree and 6 being strongly agree) please select the extent to which you agree with the above statement.

○ 1 ○ 2 ○ 3 ○ 4 ○ 5 ○ 6

Comments

Record Speech New | Record Speech Continue

## What research is NOT 2

**Routine evaluation of local authority services, for internal service monitoring and improvement, is not research.**

***For example:***

***Evaluating the impact of a community pilot scheme to determine if it should be rolled out across a local authority area.***

***Checking how a service is performing against national targets.***

On the following scale (1 being strongly disagree and 6 being strongly agree) please select the extent to which you agree with the above statement.

1 2 3 4 5 6

Comments

Record Speech New Record Speech Continue

## Final Comments

Please feel to suggest your own definition of research or provide any additional comments

Record Speech New Record Speech Continue

## Block 10

We would now like to ask you about your experience of being part of this project.

On a scale of 1 - 10 (1 meaning poor and 10 meaning excellent) please rate your experience of taking part in this project?

*You can drag the slider beneath the gauge to enter a response*

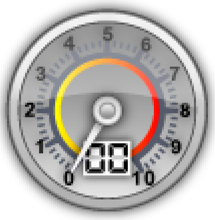


Please let us know if there is anything you feel could have been improved, or if you have any other comments about being part of the project.

Powered by Qualtrics
