# Supplementary material for: A local-authority specific definition of research: Results from a Delphi study
Source: Public Health Pract (Oxf). 2026 Mar 4;11:100765. doi: 10.1016/j.puhip.2026.100765 (PMC12996929; doi:10.1016/j.puhip.2026.100765)
Supplement: Multimedia Component 5 [file mmc5.docx]

**Definition of Applied Local Government Research- Delphi Exercise**

**Workshop Instructions**

**Summary of Group Discussion Exercise**

During the group discussion part of the workshop (40 minutes), we would like you to

work in groups to review the responses and comments from Round Two and discuss potential final consensus definitions.

**You will be split into two groups and asked to:**

- Decide whether to use all five of the Round Two definitions or whether to take the highest scoring two (definitions 1 and 2) only.
- Work on the selected definitions (as decided above) and revise as needed (reflecting panellist feedback) to arrive at some “agreed versions”.

Following the group exercise, we will come together again for an hour to hear feedback from both groups and then work collectively to agree some finalised definition statements.

The Round Two definition statements are included on page 2 of this document and contain tracked changes reflecting some of the Round Two panellist feedback.

When discussing the definitions in your groups and in the subsequent discussion, you may wish to refer to the panellist own definitions and comments as outlined in the **Delphi Round Two Responses Summary** document (and provided as separate handouts).

**Points to consider:**

- To what extent is purpose actually important in terms of the definitions (e.g. focussed on reducing inequalities) is this key to a general LA research definition?
- To what extent is generalisability actually important/relevant (some push back against this in the comments)
- Do we include what research IS NOT anywhere within the definition?

**We would also like you to consider the following:**

- Do we need to look more closely at service evaluation and what this actually means? Are we specifically including/excluding service evaluations- is it clear what we mean by this especially in a LA context?
- Should we have a blanket statement that statutory consultations are not research but everything else is?
- If we were to arrange a repository of research activity- what would/should go on it? Would this be everything including consultation or engagement or just research in a strict sense?

**Round Two Definition Statements (including tracked changes based on specific feedback).**

**Process (the ‘how’)**

1. **Using structured, organised andreproducible, methods to produce new information or knowledge, which may include testing an idea, theory, or new intervention, is research.**
2. **Using structured, organised and reproducible methods to provide a new interpretation of existing information is research. This may include routinely collected data being used for a new purpose, as well as publicly available data.**
3. **Producing findings that are generalisable (i.e. are useful beyond the original setting of the work) is research.**

**Purpose (the ‘why’)**

1. **Research is undertaken to inform decisions about practice and what policies and interventions should be implemented at a local, regional or national level.**
2. **Research seeks to help us understand how people are impacted by the context in which they live, work and go about their daily lives.**

**What research is NOT**

1. **Resident/community consultations (e.g. asking members of the public for their views) where this is considered routine practice or business as usual, is not research.**
2. **Routine evaluation of local authority services, for internal service monitoring and improvement, is not research.**
