# Supplementary material for: A local-authority specific definition of research: Results from a Delphi study
Source: Public Health Pract (Oxf). 2026 Mar 4;11:100765. doi: 10.1016/j.puhip.2026.100765 (PMC12996929; doi:10.1016/j.puhip.2026.100765)
Supplement: Multimedia Component 6 [file mmc6.docx]

**Definition of Applied Local Government Research- Delphi Exercise**

**Group Discussion**

- Decide whether to use all five of the Round Two definitions or whether to take the highest scoring two (definitions 1 and 2) only.
- Work on the selected definitions (as decided above) and revise as needed (reflecting panellist feedback) to arrive at some “agreed versions”.

**Points to consider:**

- To what extent is purpose actually important in terms of the definitions (e.g. focussed on reducing inequalities) is this key to a general LA research definition?
- To what extent is generalisability actually important/relevant (some push back against this in the comments)
- Do we include what research IS NOT anywhere within the definition?

**We would also like you to consider the following:**

- Do we need to look more closely at service evaluation and what this actually means? Are we specifically including/excluding service evaluations- is it clear what we mean by this especially in a LA context?
- Should we have a blanket statement that statutory consultations are not research but everything else is?
- If we were to arrange a repository of research activity- what would/should go on it? Would this be everything including consultation or engagement or just research in a strict sense?
