# Supplementary material for: A local-authority specific definition of research: Results from a Delphi study
Source: Public Health Pract (Oxf). 2026 Mar 4;11:100765. doi: 10.1016/j.puhip.2026.100765 (PMC12996929; doi:10.1016/j.puhip.2026.100765)
Supplement: Multimedia Component 7 [file mmc7.pdf]

NIHR | Research Support Service

Specialist Centre for Public Health

Defining research in local authorities: guidance and decision tool

Defining research in local authorities: guidance and decision tool

Contents

Chapter 1 - Consensus definition of research in a local authority context 3
Who is this document aimed at? 3
Why may you want to use this definition and decision tool? 3
The definition of research and further guidance 5
Box A: Consensus definition of research in a local authority context .... **Error! Bookmark not defined.**
Box B: Decision tool for local authority research 6
Decision tool for local authority research- process list 7
Box C: Example local authority research projects 8
Chapter 2 - Background and development of the definition of research 10
Why is a definition of research in a local authority context needed? 10
How was this definition achieved? 11
Figure 1: Outline of the process used in the Delphi exercise to reach a consensus definition. 11
Additional comments on the definition 12
Potential sources of advice 12
Working with other organisations 12
Chapter 3 - Next steps and resources 13
Research governance 14
Research governance resources 14
Research ethics 15
Research ethics resources 18
Acknowledgements 19
Appendix 1: Decision tool worked examples 22

The consensus definition (Box A), and decision tool (Box B), resulted from a formal consensus process (Delphi exercise). This exercise involved local government officers, academics and members of the public.

Chapter 1 - Consensus definition of research in a local authority context

Who is this document aimed at?

This definition and guidance document was designed by, and for, local authorities.

It provides a definition of research that you may wish to use within a local authority setting.

Why may you want to use this definition and decision tool?

You may wish to use this tool:

- **For guidance**. For example, if you are unsure if the activity you are doing, or planning to do, within a local authority is research.
- **To explore if your idea would allow you to access a resource, or would be attractive to an academic partner.** For example, determining potential eligibility for research funding or specialist support.
- **To guide activities that could be supported by** local authority research support teams or other local authority research infrastructure (for example, Health Determinants Research Collaboration (HDRC) teams).
- **To help you decide** if the activity you are planning **might need, or benefit from, independent ethical review** and formal **research governance** approval. Or if **further requirements may apply, for example, obtaining consent for research purposes**.
- **To help you determine how to classify** your activity and confirm any **associated requirements if you intend to present or publish the outputs.**

If your activity does not meet the definition of research, this does not mean that it is not worth doing. It also does not mean that there are no governance or ethical aspects to consider. You may still wish to refer to some of the guidance and resources outlined in this document.

For activities involving the NHS or adult social care, decision tools are available via the Health Research Authority (HRA) to help you determine if your activity meets the definition of research applied within these settings, and if it requires ethical review.

This definition and tool are aimed at activities happening within a local authority which are outside the scope of the HRA's work. **If your work focuses on the NHS treatment or management of a disease, clinical condition or patient group, or if it relates to adult social care activities that fall within the HRA's remit, please refer to HRA guidance instead.**

Some activities happening within local authorities, which have not traditionally been seen as ‘research’, may fully or partially meet this definition. For example, statutory consultations or resident engagement activities.

Conversely, some activities not meeting this definition may still use research skills and methods.

This document does not provide a definitive answer to the question of what ethical review or research governance processes should be applied to a local authority activity.

Nor does it aim to imply that activities, such as consultations required by law, which routinely occur in local authorities and are not normally seen as research, now need to be treated as research.

Instead, it intends to provide a starting point and signpost to further resources.

We recognise that individual local authorities will ultimately need to take their own approach to:

a) **Determining which research activities should be subject to formal research governance or independent ethical review processes.**

b) **Implementing necessary governance and ethical review processes that apply to research and ‘non-research’ activities.**

We encourage those who are unsure whether to class an activity as research, to use this guidance and to seek further advice as needed. **Further details are on page 12.**

The definition of research and further guidance

This section provides a definition of research in local authorities (Box A); a decision tool to assist with deciding whether your project meets the definition of research (Box B); and some examples of research involving local authorities (Box C).

Box A: Consensus definition of research in a local authority context

Local authority research supports decision making about practice, policies and interventions at a local, regional or national level

and/or

It helps us understand how people are impacted by the context in which they live, work and go about their daily lives.

Research uses structured, organised and reproducible methods to:

- **produce new information or knowledge**, which may include testing an idea, theory or new intervention.

  and/or

- **provide a new interpretation of existing information**. This may include routinely collected data being used for a new purpose, as well as publicly available data.

Box B: Decision tool for local authority research (a process list version of this tool is available overleaf)

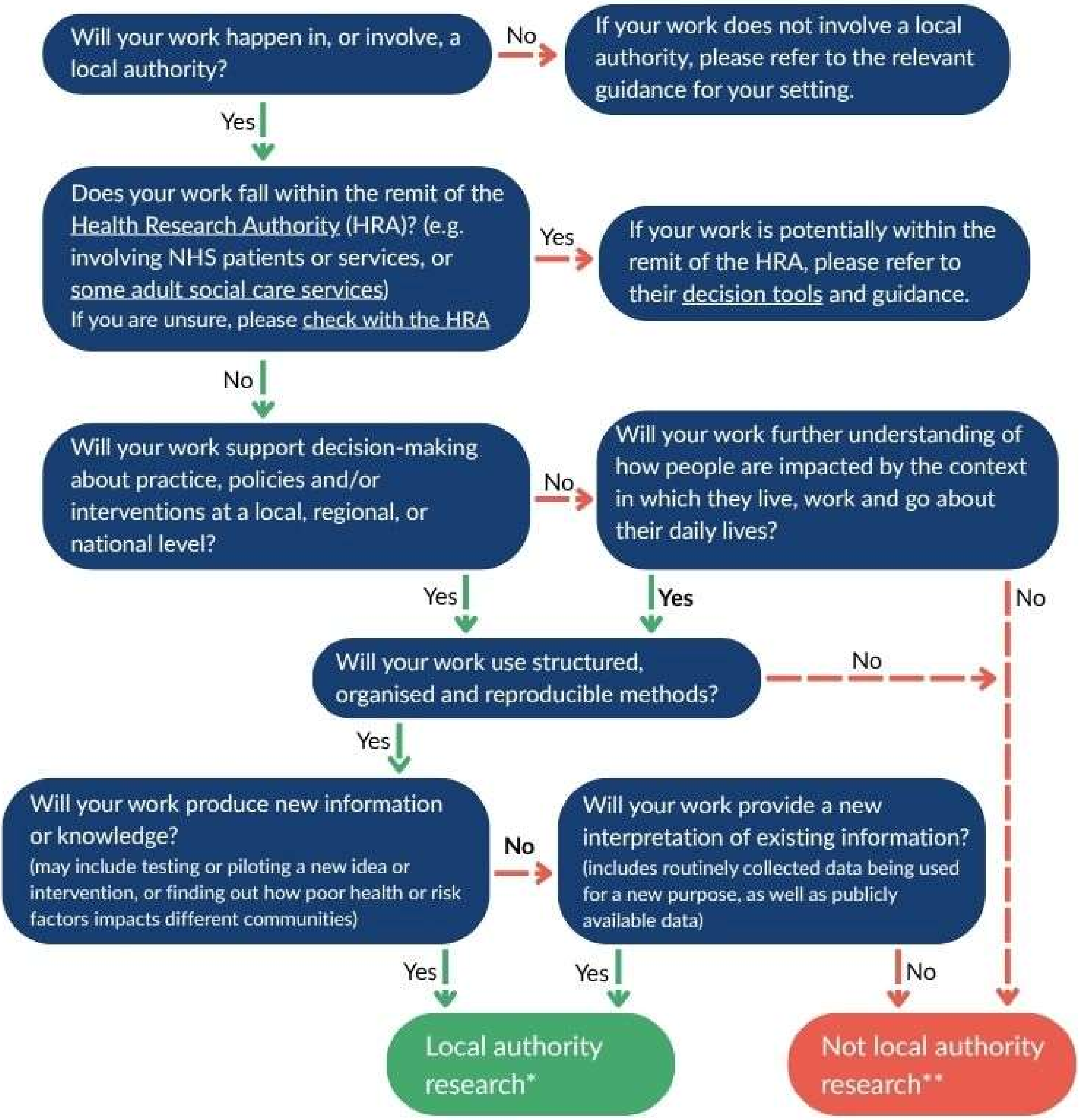

* Research as per the consensus definition of research in a local authority. Please check local advice and guidance regarding research governance and ethics processes that apply.
** Activities which are not classed as research under this tool may still be subject to local governance or ethics processes. Please check your local requirements and sources of advice and guidance.

Decision tool for local authority research- process list

1. Will your proposed work happen in, or involve, a local authority?
    a. If no, please refer to the relevant guidance for your setting.
    b. If yes, go to item 2.

2. Does your work fall within the remit of the Health Research Authority (HRA)? (e.g. involving NHS patients or services, or some adult social care services)
   If you are unsure, please check with the HRA
    a. If no, go to item 3.
    b. If yes, please refer to the Health Research Authority decision tool and guidance.

3. Will your work support decision-making about practice, policies and/or interventions at a local, regional, or national level?
    a. If no, go to item 4.
    b. If yes, go to item 5.

4. Will your work further understanding of how people are impacted by the context in which they live, work and go about their daily lives?
    a. If no, see item 8.
    b. If yes, go to item 5.

5. Will your work use structured, organised and reproducible methods?
    a. If no, see item 8.
    b. If yes, go to item 6.

6. Will your work produce new information or knowledge? (may include testing or piloting a new idea or intervention, or finding out how poor health or risk factors impacts different communities).
    a. If no, go to item 7.
    b. If yes, see item 9.

7. Will your work provide a new interpretation of existing information? (includes routinely collected data being used for a new purpose, as well as publicly available data)
    a. If no, see item 8.
    b. If yes, see item 9.

8. Not local authority research. Activities which are not classed as research under this tool may still be subject to local governance or ethics processes. Please check your local requirements and sources of advice and guidance.

9. Local authority research. Research as per the consensus definition of research in a local authority. Please check local advice and guidance regarding research governance and ethics processes that apply.

Box C: Example local authority research projects

Project Title and Reference	Description	Local authority staff roles involved	Key words
Exploring public acceptance of the use of administrative data and a co-produced local data system<br><br>NIHR Award ID: NIHR160994	Using focus groups and workshops with adults and young people to explore attitudes of the public to the sharing and wider use of their routine administrative data.	Local authority-based lead investigator (HDRC Data Manager).<br>Other local authority staff involved as co-investigators include public health consultants.	Data<br>Co-production<br>Focus-groups
Stories of life and health in an unequal society: how can local government close the health inequalities gap through a better understanding of people's experiences of resilience, coping, adversity and health in coastal communities?<br><br>NIHR Award ID: NIHR303080	The aim of this research is to understand the experiences of people living in a coastal town in West Sussex in relation to health and illness, situated within the place they live and their social context, and to engage collaboratively with coastal communities and local government to shape action to reduce coastal health inequalities which builds on local assets. Methods include participant observation, working with community researchers to undertake semi-structured interviews and walking interviews, participatory photography and participatory action research exploring citizen-led action.	Lead Investigator is a local authority Public Health Lead undertaking a PhD project as part of a Local authority fellowship.	Health inequalities<br>Interviews
Evaluation of the Zero Emission Bus Regional Area (ZEBRA) scheme: The Introduction of zero-emission buses in Oxford<br><br>NIHR Award ID: NIHR166658	Project exploring the effects of an initiative to replace diesel with electric buses. Involves collecting and using air and noise pollution data, in addition to data from interviews with stakeholders (bus operators, council staff and the public). Information on costs and cost savings will also be analysed.	Head of Place Shaping<br>Sustainable Transport Manager<br>Future Mobility and Placemaking Team Leader<br>Principal Air Quality Manager<br>Principal Infrastructure Planner	Transport<br>Air pollution<br>Mixed methods
A window of (missed) opportunity? Exploring minor-attracted adults' experiences of suicidality and mental health service interactions, to facilitate improved outcomes<br><br>NIHR Award ID: NIHR303086	Project exploring how mental health services can better understand and meet the needs of non-offending adults with child sexual attraction, to help improve their mental health and reduce the risk of offending, or suicide. Involves systematic literature review, focus groups with LA mental health practitioners, and interviews with people with lived experience.	Conducted by a Mental Health Service Manager undertaking a Doctoral Local Authority Fellowship	Literature review<br>Focus groups<br>Interviews<br>Mental health

Project Title and Reference	Description	Local authority staff roles involved	Key words
Annual Resident Survey	Annual resident survey. Around 30 questions covering ONS wellbeing questions, and questions about various domains hypothesised to impact wellbeing<br>Survey feeds into council Wellbeing Index alongside publicly available data sources + a survey specific dashboard can be used to understand population needs.	Communications (funding).<br>Survey led by Public Health; External research company contracted for data collection and descriptive statistics. Further quantitative analysis by Public Health analysts.	Survey<br>Wellbeing<br>Residents
Online Youth Safety Project	Project exploring perceptions of online safety and safety strategies among young people and adults (teachers / professionals)<br>Focus groups with young people, school staff, and parents. Joint focus groups also held between young people and school staff, Additional youth events on the topic were observed.	Led by Public Health with support from School Health and Wellbeing Manager.	Online safety<br>Young people<br>Focus groups

Non-research local authority activities may include consultations with residents or staff which are legally required and other activities which are undertaken to collect information for management purposes only.

For example:

- A survey with local residents undertaken as part of a planning application.
- A request for general feedback or comments on a planned change to a service.
- A review of key performance indicators for a commissioned service.

Chapter 2 - Background and development of the definition of research

Why is a definition of research in a local authority context needed?

High quality evidence should inform the decisions local authorities make that impact on the health and wellbeing of their local populations.

We need research to generate evidence. However, there is much variation between and within local authorities on what we mean by 'research'.

Some local authorities use a very health focussed definition, for example, the UK Policy Framework for Health and Social Care Research definition. Others use a broader definition, which includes service evaluation and consultation activities.

**Without a consistent definition of what research is, it is much harder to develop research processes and practice.**

Amongst other things:

- It creates a language barrier (when we talk about ‘research’ we don’t always mean the same thing). This makes promoting research and its benefits to colleagues and the public challenging.
- Research needs robust research governance and research ethics processes. This ensures the safety and wellbeing of everyone involved; protects organisations reputationally, financially and legally; and helps maintain public trust in research and researchers. Without an agreed definition as a starting point, identifying which activities require research governance and ethics processes, and communicating this to the relevant stakeholders, is more difficult.
- Lack of agreement on whether an activity is research can restrict access to research support or funding.

Feedback from local authorities and researchers on the need for an agreed definition prompted this work.

How was this definition achieved?

The consensus definition in Box A was developed using a formal consensus method, known as the Delphi method. It involved local authority officers covering a range of roles, departments and local authority areas within England and Scotland.

The Delphi method uses a structured approach to encourage a group of individuals towards agreement on a topic. It involves establishing a panel of experts who answer two or more anonymous questionnaire rounds, each time aiming to get closer to an agreed outcome between panellists. The panel can revise their opinions and responses as the exercise proceeds, based on feedback from previous rounds.

In this exercise, 60 local authority officers reviewed potential definitions of research. They did this via two successive rounds of questionnaires. The project Steering Committee agreed the first set of potential definitions used in the initial questionnaire, which included some recognised definitions such as the UK Policy Framework for Health and Social Care Research.

Panellists were asked to indicate if they agreed or disagreed with the potential definitions. They could also provide comments and suggest revisions.

Delphi panellists were not asked to consider the need for research governance or independent research ethics review as part of the definition. The intention was to gain consensus on an overall definition in the first instance. This could then inform further discussion around research governance and ethical review.

The responses and comments received from Round One were used to develop revised definitions for Round Two. The Round Two responses were then taken to a workshop of the Steering Committee where the final consensus definition was agreed. The process is shown in Figure 1, below.

Figure 1: Outline of the process used in the Delphi exercise to reach a consensus definition.

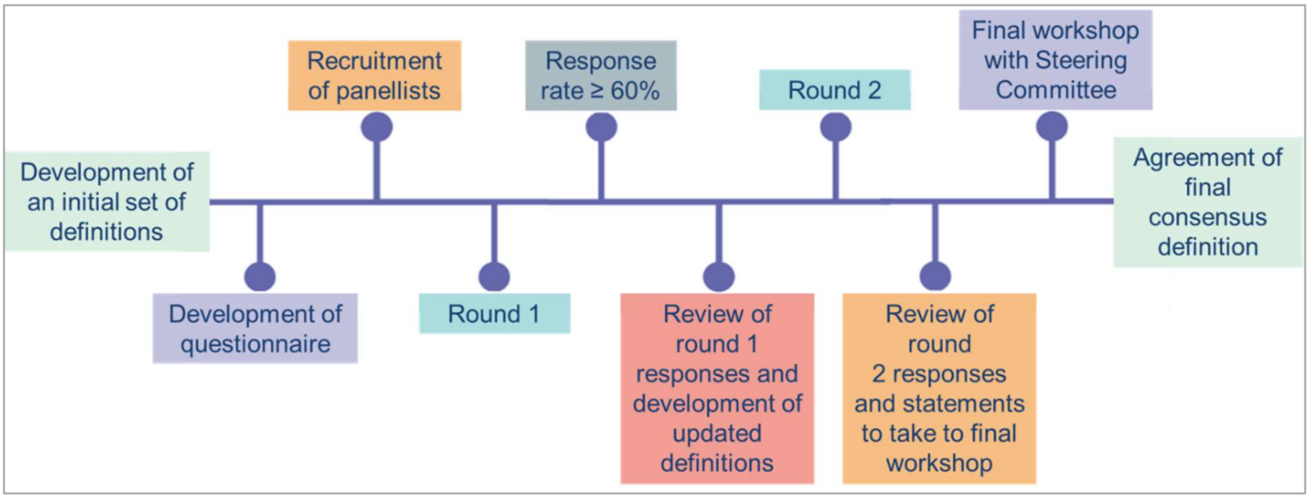

Additional comments on the definition

Deciding which local authority activities are research is not always straightforward.

There are grey areas and a range of views.

Deciding whether or not to treat something as research is not an exact science; nor is determining the level of research governance or ethical review that a research activity needs.

Potential sources of advice are:

- The NIHR Research Support Service Specialist Centre for Public Health and Specialist Centre for Social Care.
- Colleagues with research experience, such as Local Authority Research Practitioners, Public Health Engagement Leads, Embedded Researchers, Consultants in Public Health, and Directors of Public Health, colleagues within Data and Insights teams.
- NIHR Health Determinants Research Collaboration colleagues.
- Research governance and ethics support teams within the NHS or university sector.

Working with other organisations

Local authorities exist within a wider research system including universities and the NHS. These organisations have their own definitions of 'research'. They also have their own requirements for research governance and ethical review.

Local authorities should consider the requirements and processes of other organisations for research activities where they are not working alone.

We also encourage local authority colleagues to share this definition and guidance with collaborating organisations to support a joint understanding of research and potential research governance considerations within a local authority setting.

Regardless of the processes and approvals within other organisations local authorities will need to decide whether their involvement in a research activity is appropriate and appropriately managed.

Chapter 3 - Next steps and resources

There are activities that will fall within the consensus definition of research, which may not require, or benefit from, independent ethical review or detailed research governance processes.

We do not propose that local authorities automatically require independent ethical review, or formal research governance approval, for all activities that meet this definition.

Rather, we intend that this definition and associated decision tool will act as a starting point to inform local authority decision making.

We note that governance and ethical requirements will also form part of usual practice and that there will be legal, ethical and professional standards that apply regardless of whether an activity is classed as research or not. You may wish to refer to any relevant resources, professional standards and guidelines when planning your activity.

We have included some information below on research governance and research ethics and a list of things you may want to consider when thinking about whether, and what, processes are required. A list of useful resources and further reading on these topics is also provided.

In addition, we would recommend consulting any relevant policies within your organisation.

Research governance

Research governance is about ensuring that research activities are safe, appropriate, legal, transparent, adequately insured and in-line with organisational policies, processes and values.

Local authorities may be involved in research in various ways and may be working with different organisations on research activities where each organisation will have their own research governance processes and priorities.

The extent and details of the local authority-specific research governance processes required will depend on the type of research and the role of the local authority within the project.

In any circumstances, processes will be needed by a local authority to:

- Confirm what they are responsible for, and what the responsibilities of other organisations involved are.
- Determine who within the authority is accountable for discharging these responsibilities, how they will meet them (including being able to demonstrate this) and how these fit with other local authority legal and ethical responsibilities.
- Manage any risks that may arise.

Research governance resources

NIHR RSS SCPH Research Governance Explainer

UK Research Integrity Office (UKRIO) Code of Practice for Research

Guidance for reviewers of research applications based in settings outside of the NHS

Kolstoe, S.E. and Pugh, J. (2024) 'The trinity of good research: Distinguishing between research integrity, ethics, and governance', Accountability in Research, 31(8), pp. 1222-1241. Available at: https://doi.org/10.1080/08989621.2023.2239712

Research ethics

Taking an ethical approach and ensuring that you have an ongoing regard for ethical research principles should be a core principle throughout any research activity.

As part of this, a key question for any proposed research projects, however, is whether formal independent ethical review is required.

Ethical review of a research project focusses on the ethical acceptability of the proposed project and on any ethical issues that may be associated with it.

For certain activities, independent ethical review by people external to your project or organisation may not be needed. You should always check with any organisations involved to confirm if they have a policy that suggests otherwise.

This is often the case for the following activities:

- Projects using existing data that is completely anonymised (where individuals are not directly or indirectly identifiable).
- Projects using some types of information that are publicly available.

Just because something is publicly available does not mean that there are no ethical considerations for its use in research. For example, there are often ethical considerations when using data from online sources such as social media and therefore need for ethical review may be determined by the type of information, its source and intended use.

For some activities, independent ethical review may be a requirement of a partner organisation, funder, in legislation, or by a regulatory body.

The following can help you determine if independent ethical review is required:

- In England and Wales, under the Mental Capacity Act 2005, you are legally required to seek approval from an appropriate body if you are undertaking intrusive research, defined as any activity where consent would legally be required for that activity, with adults who are unable to provide consent. If you are in Scotland and Northern Ireland, you should check the relevant national legislation for ethical review requirements for adults lacking capacity.
- If your project involves the NHS, or users of adult social care, the HRA decision tool can be used to determine requirements for ethical review.
- If you are working with a university, the university will usually have a clear policy on ethical review requirements. You can check with your university colleagues about this.
- If you are in receipt of external funding from a research funding body or other organisation, requirement for independent ethical review may be within your funding terms and conditions.

In other circumstances, the need for, or benefit from having, independent ethical review can be less clear.

In such circumstances, you may want to consider the following:

- Will you be working with children or [vulnerable](#) adults, or those who could be considered vulnerable in the context of your research?
- Will you be accessing or using [sensitive information](#) about people?
- Will your research involve any sensitive, controversial or potentially distressing issues or topics?
- Are you planning to involve people in your research with whom you have an existing professional or personal relationship. For example, are you a practitioner whose research will involve existing clients or service users?
- Will you be approaching people to take part in your research, or accessing information for your research, via a third party (this is often called a gatekeeper)?
- What will you be asking people to 'do' as part of your research? Does it involve any potential risks to their safety or welfare, or could it be potentially burdensome, or time-consuming for them?
- Does your research involve providing or withholding an intervention or implementing a change to routine practice or service provision? Is this intervention or change being offered or withheld based on certain criteria, or selection processes, or being offered for a limited time only?
- Will you be deceiving or withholding information from people in some way?

If the answer to any of the above is yes, you may want to consider seeking independent ethical review.

This is especially the case if you are planning to publish your research in an academic journal. Journal editors may wish to see evidence of independent ethical review for certain types of project, or may require justification for not seeking ethical review prior to publication.

For some projects, ethical review may already have been undertaken by another organisation. Where this is the case, you should check with your local authority if further ethical review is needed. There may be circumstances where it is appropriate for university or NHS ethical review to be accepted by a local authority, however there may also be circumstances where a local authority requires its own ethical review process to be followed.

If you feel that independent ethical review would be helpful, but are unsure where to go, or how to access a review, the sources of advice listed on page 12 will be able to suggest options.

Research ethics resources

NIHR RSS SCPH Research Ethics Explainer

UK Research Integrity Office (UKRIO) Research Ethics Support and Review

Government Social Research Ethics Checklist

UK Statistics Authority Ethics Guidance Documents

Social Research Association Research Ethics Guidance

Kolstoe, S.E., Sözüdoğru, E., Messer, J., Coates, E. and Tobin, E. (2025) 'Is my project research? Determining which projects require review by a research ethics committee', *Accountability in Research*, pp. 1-21. Available at: https://doi.org/10.1080/08989621.2025.2460521

Levitas, A., Taylor, E., Navelle, P.L., Humphreys, E. and Sheringham, J. (2025) 'Local authorities need tailored research ethics processes to support research capacity building', *Public Health in Practice*, 9, p. 100587. Available at: https://doi.org/10.1016/j.puhip.2025.100587

Acknowledgements

This project was funded by the NIHR Research Support Service Specialist Centre for Public Health.

This guidance has been developed by the Definition of Research in Local Authorities project Steering Committee and is published by the NIHR Research Support Service Specialist Centre for Public Health (NIHR RSS SCPH).

The members of the Steering Committee from outside the NIHR RSS Specialist Centre for Public Health contributed to this project and guidance in an individual capacity and not as representatives of their organisations.

We thank the project Steering Committee, NIHR RSS SCPH colleagues, and the many local authority colleagues who have been involved in the definition of research project and in advising on the content and format of this guidance. Particular thanks go to Kay Howes for initially convening the Steering Committee, Charlotte Rothwell for her advice on the Delphi method, and to Alex Rodger and Chantel Davies for their input on the design and accessibility of this guidance.

Please cite this guidance as:

Brown, L., Sowden, S., Briggs, A., Dennington-Price, A., Dutta, V., Hampshaw, S., Humphreys, E., Johns, T., Levitas, A., Murphy, R., Needham, K., Renwick, L., Hayes, L. (2025) *Defining research in local authorities: guidance and decision tool.* Available at: URL (Accessed: date)

Steering Committee membership

Dr Sarah Sowden (Chair)
Senior Clinical Lecturer, Newcastle University
Consultant in Public Health, Office for Health Improvement and Disparities
Deputy Director, NIHR RSS Specialist Centre for Public Health delivered by Newcastle University and partners

Professor Adam Briggs
Deputy Director of Public Health, Oxfordshire County Council
Deputy Director, NIHR RSS Specialist Centre for Public Health delivered by Southampton University and partners
Director, NIHR Public Health Research Programme

Laura Brown
Research Programme Manager, NIHR RSS Specialist Centre for Public Health delivered by Newcastle University and partners

Abi Dennington-Price
Patient & Public Representative

Dr Vanita Dutta
Public Health Principal, Slough Borough Council

Dr Susan Hampshaw
Director, Health Determinants Research Collaboration (HDRC) – Doncaster
City of Doncaster Council

Dr Louise Hayes
Research Design Lead, NIHR RSS Specialist Centre for Public Health delivered by Newcastle University and partners

Emily Humphreys
Consultant in Public Health, London Borough of Tower Hamlets
Director, Health Determinants Research Collaboration (HDRC) – Tower Hamlets

Tracey Johns
Institute of Public Health and Wellbeing, University of Essex
Public & Community Partnerships Research Adviser, NIHR RSS Specialist Centre for Public Health delivered by Newcastle University and partners

Alexandra Levitas
Embedded Researcher for Evidence Islington, London Borough of Islington

Rachel Murphy
Service Manager- Research Development, North Yorkshire Council
Health Determinants Research Collaboration (HDRC) – North Yorkshire

Katie Needham
Consultant in Public Health, North Yorkshire Council
Director, Health Determinants Research Collaboration (HDRC) – North Yorkshire

Laura Renwick
Public Health Senior Research Manager, NIHR RSS Specialist Centre for Public Health delivered by Newcastle University and partners

Appendix 1: Decision tool worked examples

Example 1: Repositioning of foods in a school canteen

Proposed activity:

Repositioning food and drinks to increase the accessibility of healthy foods and decrease the accessibility of unhealthy foods within a local authority school setting.

The aim of the project is to determine whether creating a healthier eating environment in this way could be an effective strategy for promoting healthier eating behaviours which should be adopted across schools within the area.

Unidentifiable purchasing data from the two schools involved in the project will be collected for set time-periods before, during and after the intervention.

The amounts of healthy and unhealthy food items purchased during the selected time periods will be analysed using recognised statistical techniques.

Worked example using the decision tool:

1. Will your work happen in, or involve, a local authority?
   Yes- the project will take place within a local authority school setting.

   Go to item 2

2. Does your work fall within the remit of the Health Research Authority? (e.g. involving NHS patients or services, or some adult social care services)
   No- NHS patients, services and adult social care services are not involved.

   Go to item 3

3. Will your work support decision-making about practice, policies, or interventions at a local, regional, or national level?
   Yes- the aim of the project is to determine if creating a healthier eating environment in this way could be an effective strategy for promoting healthier eating behaviours which should be adopted across schools within the area.

   Go to item 5

5. Will your work use structured, organised and reproducible methods?
   Yes- the amounts of healthy and unhealthy food items purchased during the selected time periods were analysed using recognised statistical techniques.

<u>Go to item 6</u>

6. Will your work produce new information or knowledge?
   Yes- information about purchasing of certain foods before, during and after the intervention periods will be produced.

   <u>Go to item 7</u>

7. Will your proposed work use structured, organised and reproducible methods?
   Yes- the amounts of healthy and unhealthy food items purchased during the selected time periods were analysed using recognised statistical techniques.

   <u>See item 9</u>

9. **This is local authority research** as per the consensus definition of research in a local authority. Please check local advice and guidance regarding the research governance and research ethics processes that apply.

Decision tool pathway

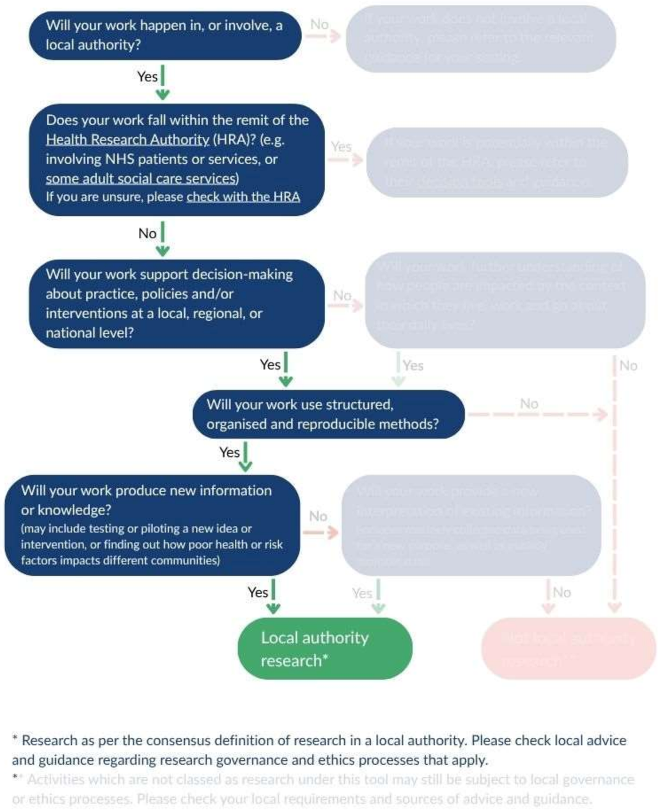

Example 2: Local authority open spaces provision assessment

Proposed activity:

Review of open spaces provision within a local authority area.

A survey on local needs and a review of existing information and consultation around open spaces will be conducted.

An audit of local open space provision will be conducted.

The project will provide the council with up-to-date information on green space location, coverage and provision.

Worked example using the decision tool:

1. Will your work happen in, or involve, a local authority?
   Yes- the project will take place within a local authority area.

   Go to item 2

2. Does your work fall within the remit of the Health Research Authority? (e.g. involving NHS patients or services, or some adult social care services)
   No- NHS patients, services and adult social care services are not involved.

   Go to item 3

3. Will your work support decision-making about practice, policies, or interventions at a local, regional, or national level?
   Yes- the project will provide the council with up-to-date information on green space location, coverage and provision.

   Go to item 5

5. Will your work use structured, organised and reproducible methods?
   Yes- the project may use methods including a survey and audit.

   Go to item 6

6. Will your work produce new information or knowledge
   No- the project will provide the council with up-to-date information on green space location, coverage and provision (but will not provide anything 'new').

   Go to item 7

7. Will your work provide a new interpretation of existing information? This includes routinely collected data being used for a new purpose, as well as publicly available data.
   No- the project will provide the council with up-to-date information on green space location, coverage and provision (but will not re-interpret or provide anything 'new').

   See item 8

8. **This is not local authority research.** Activities which are not classed as research under this tool may still be subject to local governance or ethics processes.

Decision tool pathway

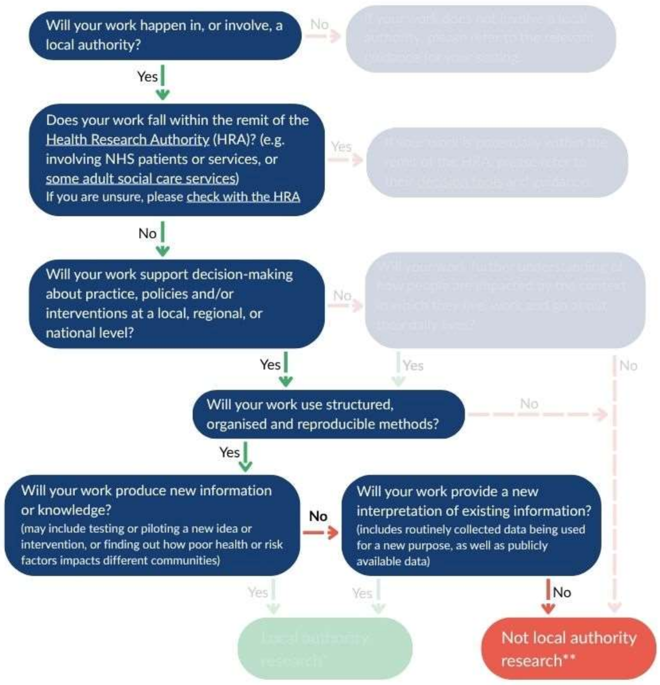

* Research as per the consensus definition of research in a local authority. Please check local advice and guidance regarding research governance and ethics processes that apply.
** Activities which are not classed as research under this tool may still be subject to local governance or ethics processes. Please check your local requirements and sources of advice and guidance.
